# Impact of Emerging Health Insurance Arrangements on Diabetes Outcomes and Disparities: Rationale and Study Design

**DOI:** 10.5888/pcd10.120147

**Published:** 2013-01-31

**Authors:** J. Frank Wharam, Steve Soumerai, Connie Trinacty, Emma Eggleston, Fang Zhang, Robert LeCates, Claire Canning, Dennis Ross-Degnan

**Affiliations:** Author Affiliations: Steve Soumerai, Connie Trinacty, Emma Eggleston, Fang Zhang, Robert LeCates, Claire Canning, Dennis Ross-Degnan, Harvard Medical School and Harvard Pilgrim Health Care Institute, Boston, Massachusetts.

## Abstract

Consumer-directed health plans combine lower premiums with high annual deductibles, Internet-based quality-of-care information, and health savings mechanisms. These plans may encourage members to seek better value for health expenditures but may also decrease essential care. The expansion of high-deductible health plans (HDHPs) represents a natural experiment of tremendous proportion. We designed a pre–post, longitudinal, quasi-experimental study to determine the effect of HDHPs on diabetes quality of care, outcomes, and disparities. We will use a 13-year rolling sample (2001–2013) of members of an HDHP and members of a control group. To reduce selection bias, we will limit participants to those whose employers mandate a single health insurance type. The study will measure rates of monthly hemoglobin A1c, lipid, and albuminuria testing; availability of blood glucose test strips; and rates of retinal examinations, high-severity emergency department visits, and preventable hospitalizations. Results could be used to design health plan features that promote high-quality care and better outcomes among people who have diabetes.

## Introduction

As discussed by Gregg et al in an accompanying article in this issue of *Preventing Chronic Disease* (1), diabetes is a growing threat to public health. In addition to its detrimental clinical impacts, diabetes creates an economic burden on both people and the health care system. Because type 2 diabetes and other chronic diseases are associated with both rising costs and modifiable lifestyle factors, consumer-directed health care advocates suggest that health systems should encourage greater patient cost-awareness and individual responsibility for health (2,3). They theorize that providing patients with information about health care quality while exposing them to full costs will create “activated health care consumers” (3). More than a decade ago, managed care organizations began to implement this theoretical framework in the form of “consumer-directed health plans” (4). These arrangements typically combine high-deductible health plans (HDHPs), Internet-based quality-of-care information, and mechanisms for saving money toward health expenses (5). Annual deductibles for the most rapidly growing HDHPs (health savings account–eligible plans [HSAs]) range from $2,400 to $12,100 per family (6,7). Advocates theorize that not only will HDHP members seek low-cost, high-quality care but they will also be more likely to adopt healthy behaviors to reduce future costs (2,3). For example, patients with diabetes may improve their diets, exercise regimens, and adherence to drugs and routine monitoring.

The expansion of HDHPs represents a natural experiment of tremendous proportion. Membership tripled between 2006 and 2012 (7), and 34% of US workers now have HDHPs (7). The rapid growth in HDHPs has been accompanied by concern — based on studies such as the RAND Health Insurance Experiment (8) — that high cost-sharing may reduce appropriate as well as inappropriate use. Recent evidence suggests that when necessary care such as essential medications (9–11) and screening tests (12,13) are subject to deductibles, use decreases. A newer school of thought has promoted “value-based insurance” designs as a remedy (14). These plans seek to broadly control costs using high deductibles while preserving evidence-based care through financial incentives. For example, plans may selectively exempt preventive visits or hypoglycemic drugs from full cost sharing. Most HDHPs now have some value-based design features (7).

Despite their rapid expansion, the fundamental hypotheses of consumer-directed health plans with value-based features have largely been untested. Among diabetic populations, excluding secondary preventive services from cost sharing may either preserve use or lead to only small declines (15–18). One study found that both high- and low-income HDHP members with diabetes experienced small decreases in appropriate diabetes care (17). However, most studies have not controlled for member-level selection or examined adverse clinical outcomes. Furthermore, no studies have compared the effect of HDHPs with and without full prescription drug cost sharing on diabetes outcomes.

Our investigation seeks to determine the effect of HDHPs on diabetes quality of care, outcomes, and disparities. We are using a longitudinal, national data set that includes 2 million members with diabetes. We have 2 primary objectives:

1. To determine the effect of HDHPs on diabetes monitoring and clinical outcomes (including high-severity emergency department visits, preventable hospitalizations, and hospitalization days) in a national population and among people from vulnerable subgroups (blacks, Hispanics, those of low socioeconomic status, and high-morbidity patients with diabetes).
2. To determine the effect of HDHPs with and without full drug cost sharing on rates of medication adherence and related clinical outcomes, both overall and among high-risk subgroups.

## Study Design

We will identify a 13-year rolling sample (2001–2013) of HDHP members and members of a control group insured by a large national health plan. Preliminary analyses indicate that the pool of commercially insured persons from which we will select our sample is closely representative of the privately insured US population by age and sex (19). Our preliminary data set (2000–2009) includes 1.3 million members aged 18 to 64 years (2.5% aged 18–24, 31.0% aged 25–39, and 66.5% aged 40–64) with predominantly type 2 diabetes. Most members reside in the South (50.3%) and Midwest (28.8%), and 48% are women. Our data source can be linked to member-level sociodemographic variables, which provide self-reported information about disposable income, home ownership, and net worth. We will also use geocoded variables on socioeconomic status. Data on race/ethnicity, derived from a combination of surname analysis and geocoded census data, are provided in the preliminary data set. Approximately 1% have missing data in 2009 for education level, income, net worth, and race/ethnicity. Most members with reported race/ethnicity data are white (75.0%); 9.8% are black, and 11.5% are Hispanic. Overall, 40.0% of members are in health maintenance organizations (HMOs), 18.3% are in preferred-provider organizations (PPOs), and 29.1% are in point-of-service (POS) health plans; 15.5% were in an account-based HDHP at some point during their enrollment. 

We will use a pre–post, longitudinal, quasi-experimental study design, a rigorous retrospective approach that we have used in previous HDHP studies (12,13,18,20). Our eligible cohort will consist of members enrolled in traditional plans (HMO, PPO, or POS) with low deductibles ($250 or less individual deductible amount) for at least 1 year who experience an employer-mandated switch to an HDHP (ie, employees have no choice in selecting the type of health plan coverage). We will follow people for 1 year before and up to 3 years after the date of this mandated transition. Our comparison group will comprise contemporaneously enrolled members whose employers chose to remain in traditional low-deductible plans during the same period and who also were offered no choice of plan by employers. Including only members with mandated insurance coverage reduces the potential for bias resulting from individual self-selection into HDHPs. In selecting study groups, we will use employer-level propensity score matching to reduce differences between HDHP and traditional employers and member-level propensity score matching to reduce residual confounding. Propensity score matching is an established method for selecting a control group with a similar likelihood as the intervention group of selecting an intervention (in this case, choosing an HDHP) on the basis of observed characteristics when people have not been randomly allocated into study groups (21–23).

## Planned Study Outcomes

Our study will focus on the clinical effects of HDHPs. To assess changes in disease monitoring, we will measure rates of monthly hemoglobin A1c, lipid, and urine microalbumin testing (but not changes in test results because there is low completeness of lab value data); use of blood glucose test strips; and rates of retina examinations. We will graphically depict these outcomes using patient-level interrupted time series with comparison series plots. Using segmented longitudinal models (18), we will estimate changes in level and trend in use after the date of the switch to HDHPs, while controlling for autocorrelation and individual-level covariates using generalized estimating equations (24). Our clinical outcomes will include annual rates of high-severity emergency department visits, preventable hospitalizations, and inpatient hospital days. We will analyze these less frequent outcomes using a difference-in-differences approach with generalized linear models. Difference-in-differences calculations involve subtracting follow-up-minus-baseline rates for the control group from follow-up-minus-baseline rates for the intervention group. Therefore, the effect of the intervention is adjusted both for the intervention group’s baseline rates and the control group’s change. This is the most rigorous retrospective approach available for estimating changes in low-frequency outcomes when time-series plots are unstable. For all analyses, we will stratify the population into vulnerable and less vulnerable groups when analyzing the effects of HDHPs on underserved populations, such as people who have low socioeconomic status.

To examine the effect of differential drug cost sharing, we will examine 2 measures of medication availability: the average number of oral hypoglycemic or antihypertensive medications available each month and the proportion of days that members have lipid-lowering or primary oral hypoglycemic medication available per month (“proportion of days covered”) (18). We will use an interrupted time-series design to compare changes in level and trend of medication use between the study groups. We will subsequently stratify analyses to determine whether HDHPs that have more generous medication coverage are associated with more favorable emergency department and hospital outcomes.

## Methodological Decision Making and Limitations

A key design decision by our research team was to restrict the sample to health plan members who have no choice of health plan, which has the disadvantage of restricting the study to smaller employers who tend to not offer insurance choices. It also precludes the ability to examine different patterns of use between members who self-select HDHPs versus those who are required to enroll. However, removing members with a choice of plans has the substantial advantage of minimizing self-selection bias, a threat to validity in health insurance studies. We also recognize that employers may choose health plans on the basis of the characteristics of their workers, such as anticipated health needs or trends in costs; for this reason, we will use propensity score matching to reduce differences between HDHP members and traditional plan members and, when possible, use time-series plots and interrupted time-series analyses, which will demonstrate whether follow-up trends are different from baseline trends. A limitation of all health insurance claims-based studies is that members drop from the sample for reasons such as losing insurance, changing jobs, or changing insurer. We will choose only members who were continuously enrolled for a full 2 years. This approach removes bias due to differential dropout between groups, but there is also risk that members with longer continuous enrollment will have unusual characteristics, limiting generalizability. Preliminary calculations indicate that 49% of our sample will have 2 full years of continuous enrollment. Finally, some members in our cohort will be eligible to serve as either an HDHP member or a control, if, for example, they had 2 years of traditional plan enrollment followed by a year in an HDHP. We are therefore validating a method of randomizing such members to either the HDHP or control group.

## Implications for Policy Makers and Clinicians

In the context of continuing rapid growth of HDHPs, results from our study can be used to design health plans that promote high-quality care and better outcomes among diabetic populations (25). Policy makers could use findings to identify tests and therapies that should be exempt from full cost sharing, potentially informing changes to account-based HDHPs and facilitating extensions of value-based insurance design. Results also may affect the health plan arrangements that regulators include in emerging state-based health insurance exchanges. For example, evidence that exempting hypoglycemic drugs from full cost sharing preserves appropriate use may make this a standard or mandated arrangement.

We will present findings at research and policy conferences attended by policy makers and clinicians. We will also meet directly with public and private policy makers at the Centers for Medicare and Medicaid Services, the Centers for Disease Control and Prevention (CDC), the Agency for Healthcare Research and Quality, and private insurance associations, among others, to discuss the implications of our research for policy decisions.
